# An Antagonism Between Ethylene Signaling and DNA Methylation Orchestrates the Progression of Leaf Senescence in Non‐heading Chinese Cabbage

**DOI:** 10.1002/advs.202414954

**Published:** 2025-06-23

**Authors:** Dingyu Zhang, Yong Luo, Han Wang, Xiaofeng Li, Liming Miao, Lu Gao, Haibo Hao, Xinman Wang, Benke Kuai, Hongfang Zhu, Guodong Ren

**Affiliations:** ^1^ Shanghai Key Laboratory of Protected Horticultural Technology Horticultural Research Institute Shanghai Academy of Agricultural Sciences Shanghai 201403 China; ^2^ State Key Laboratory of Genetics and Development of Complex Phenotypes Fudan Center for Genetic Diversity and Designing Agriculture School of Life Sciences Fudan University Shanghai 200438 China; ^3^ State Key Laboratory for Protein and Plant Gene Research Peking‐Tsinghua Joint Center for Life Sciences School of Life Sciences Peking University Beijing 100871 China

**Keywords:** CMT2, DNA methylation, EIN3A, ethylene, leaf senescence, non‐heading Chinese cabbage

## Abstract

Leaf senescence significantly impacts both the yield and quality of non‐heading Chinese cabbage (NHCC). Despite its importance, the molecular mechanisms underlying the initiation and progression of leaf senescence in NHCC remain poorly understood. Here, transcriptome changes are first profiled, and found that ethylene (ET) and salicylic acid (SA) signaling pathways are activated during leaf senescence. Further mining of ET, SA, and DNA methylation pathway genes identified that *EIN3A* and *CMT2* are induced and repressed, respectively, during leaf senescence. A whole‐genome bisulfite sequencing (WGBS) analysis revealed a global reduction of DNA methylation during leaf senescence, with the most dramatic decline occurring in the 1 kb regions upstream of transcription start sites (TSS) and downstream of transcription end sites (TES). Knocking down of *EIN3A* and *CMT2* via virus‐induced gene silencing (VIGS) demonstrated that EIN3A is crucial for ET‐ and age‐triggered leaf senescence, while CMT2 plays an inhibitory role. It further showed that EIN3A directly represses the expression of *CMT2* to release its “braking” role on senescence‐associated genes (*SAGs*) expression. Thus, the study uncovers a pivotal antagonistic interaction between ET signaling and DNA methylation, which may be involved in setting the pace for the progression of leaf senescence in NHCC.

## Graphical abstract

CMT2 mediates the maintenance of DNA methylation on the promoters of *SAGs*, thereby inhibiting EIN3A untimely activating *SAGs*’ expression. Aging‐impelled striking upregulation of *EIN3A* strongly suppresses the expression of *CMT2* to reduce the methylation level, which releases the activation of EIN3A on *SAGs*, thus initiating and accelerating the progression of leaf senescence in NHCC.

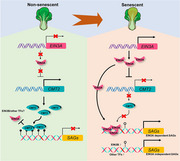

## 1 Introduction

Leaves are major photosynthetic organs that convert light energy into chemical energy. However, during senescence, leaves shift their roles by actively breaking down macromolecular nutrients and reallocating them to the developing organs to support their growth.^[^ ^1^ ^]^ In grain crops where seeds are the target of harvest, the remobilization of macromolecular nutrients from senescing leaves serves as a vital “source” of nutrients.^[^ ^2^ ^]^ In contrast, in vegetable crops where leaves are the harvested organ, leaf senescence incurs direct losses in both yield and quality, markedly reducing their economic and nutritional value.^[^ ^3^ ^]^ Therefore, deciphering the molecular mechanisms of leaf senescence in different plant species holds distinct biological and agronomic importance.

The initiation and progression of leaf senescence are mediated by phytohormones, which integrate both intrinsic and extrinsic signals to modulate leaf senescence.^[^ ^1^, ^4^ ^]^ Ethylene (ET) is the most crucial phytohormone in regulating leaf senescence and fruit ripening.^[^ ^5^ ^]^ In‐depth studies on ET‐regulated leaf senescence and fruit maturation have primarily utilized model plant species such as *Arabidopsis thaliana* and *Solanum lycopersicum*.^[^ ^1^, ^6^ ^]^ The process of ET‐regulated leaf senescence involves a molecular module that includes ETHYLENE INSENSITIVE 2 (EIN2), ETHYLENE INSENSITIVE 3 (EIN3), a NAC transcription factor ORESARA1 (ORE1), and *miR164*, with *ORE1* being targeted and degraded by *miR164*.^[^ ^7^ ^]^ The transcription factor EIN3, located downstream of EIN2, directly regulates the expression of *miR164* and *ORE1*.^[^ ^8^ ^]^ Our previous research showed that EIN3 and its target ORE1 cooperatively activate the expression of chlorophyll catabolic genes (*CCGs*) to trigger chlorophyll degradation.^[^ ^8a^ ^]^ Moreover, EIN3 also interacts with NONEXPRESSOR OF PATHOGENESIS‐RELATED GENES 1 (NPR1), mediating the crosstalk of salicylic acid (SA) and ET on regulating leaf senescence.^[^ ^9^ ^]^ Furthermore, dysfunction in EIN3 significantly delays dark‐ and methyl jasmonate (MeJA)‐induced leaf senescence.^[^ ^8b^ ^]^ Thus, EIN3 acts as a key hub integrating multiple signals to trigger leaf senescence. It is noteworthy that, apart from leaf senescence, EIN3 also regulates various physiological/developmental processes, and the precise mechanisms by which EIN3 recognizes and targets specific responsive genes at different developmental stages still remain largely unclear.^[^ ^10^ ^]^

DNA methylation, a conserved epigenetic modification in most eukaryotes, influences numerous developmental and stress‐responsive processes through affecting gene expression and/or maintaining genome stability.^[^ ^11^ ^]^ The level of DNA methylation is modulated by the balanced action of DNA methyltransferases and demethylases. In Arabidopsis, DOMAINS REARRANGED METHYLASE 2 (DRM2) is the major methyltransferase that catalyzes *de novo* DNA methylation through the RNA‐directed DNA methylation (RdDM) pathway.^[^ ^12^ ^]^ For maintenance of DNA methylation, METHYLTRANSFERASE 1 (MET1) is responsible for CG methylation, while CHROMOMETHYLASE 2 (CMT2) and CMT3 catalyze CHG methylation. Additionally, asymmetric CHH methylation is catalyzed by CMT2 and/or DRM2, depending on chromatin context. On the other hand, active DNA demethylation is mediated by bifunctional 5mC DNA glycosylases, including REPRESSOR OF SILENCING 1 (ROS1), DEMETER‐LIKE PROTEIN (DML), and TRANSCRIPTIONAL ACTIVATOR DEMETER (DME).^[^ ^11^, ^13^ ^]^ Several studies have highlighted the role of active DNA demethylation in regulating leaf senescence and fruit ripening.^[^ ^13^ ^]^ For instance, the Arabidopsis DNA demethylase *DML3* is significantly upregulated in senescent leaves, promoting active DNA demethylation, thus affecting the expression of hundreds of senescence‐associated genes (*SAGs*) to accelerate leaf senescence.^[^ ^14^ ^]^ Similarly, a decline in DNA methylation, triggered by DML2‐mediated active cytosine demethylation or downregulation of RdDM‐mediated *de novo* DNA methylation, is associated with fruit ripening in tomatoes and strawberries.^[^ ^15^ ^]^ These findings underscore the importance of global DNA demethylation during leaf senescence and fruit ripening.

Non‐heading Chinese cabbage (NHCC, *Brassica rapa* ssp. *chinensis*), which includes many varieties such as pak choi (*Brassica rapa* ssp*. chinensis* var. *communis*), caixin (*Brassica rapa* ssp*. chinensis* var*. parachinensis*), fenniecai (*Brassica rapa* ssp*. chinensis* var. *nipposinica*), and tacai (*Brassica rapa* ssp. *chinensis* var. *rosularis*), is an important vegetable crop being widely cultivated, especially in Asia.^[^ ^16^ ^]^ The rate of leaf senescence is a critical trait impacting the yield and quality of NHCC. However, the molecular mechanisms underlying leaf senescence in NHCC remain largely unexplored.^[^ ^17^ ^]^ In this study, we profiled the global transcriptome and DNA methylome changes during leaf senescence in NHCC and discovered that EIN3A and CMT2 constitute an antagonistically cooperative module in orchestrating ET‐ and age‐triggered leaf senescence in NHCC. EIN3A is induced during leaf senescence and directly inhibits the expression of *CMT2*, which reduces DNA methylation and releases EIN3A's activation of certain *SAGs*. Our results provide new insights into the coordination between transcription factors and epigenetic modifications in modulating leaf senescence in NHCC.

## 2 Result

### 2.1 Physiological and Transcriptional Analysis of Natural Leaf Senescence in NHCC

**Figure 1:**
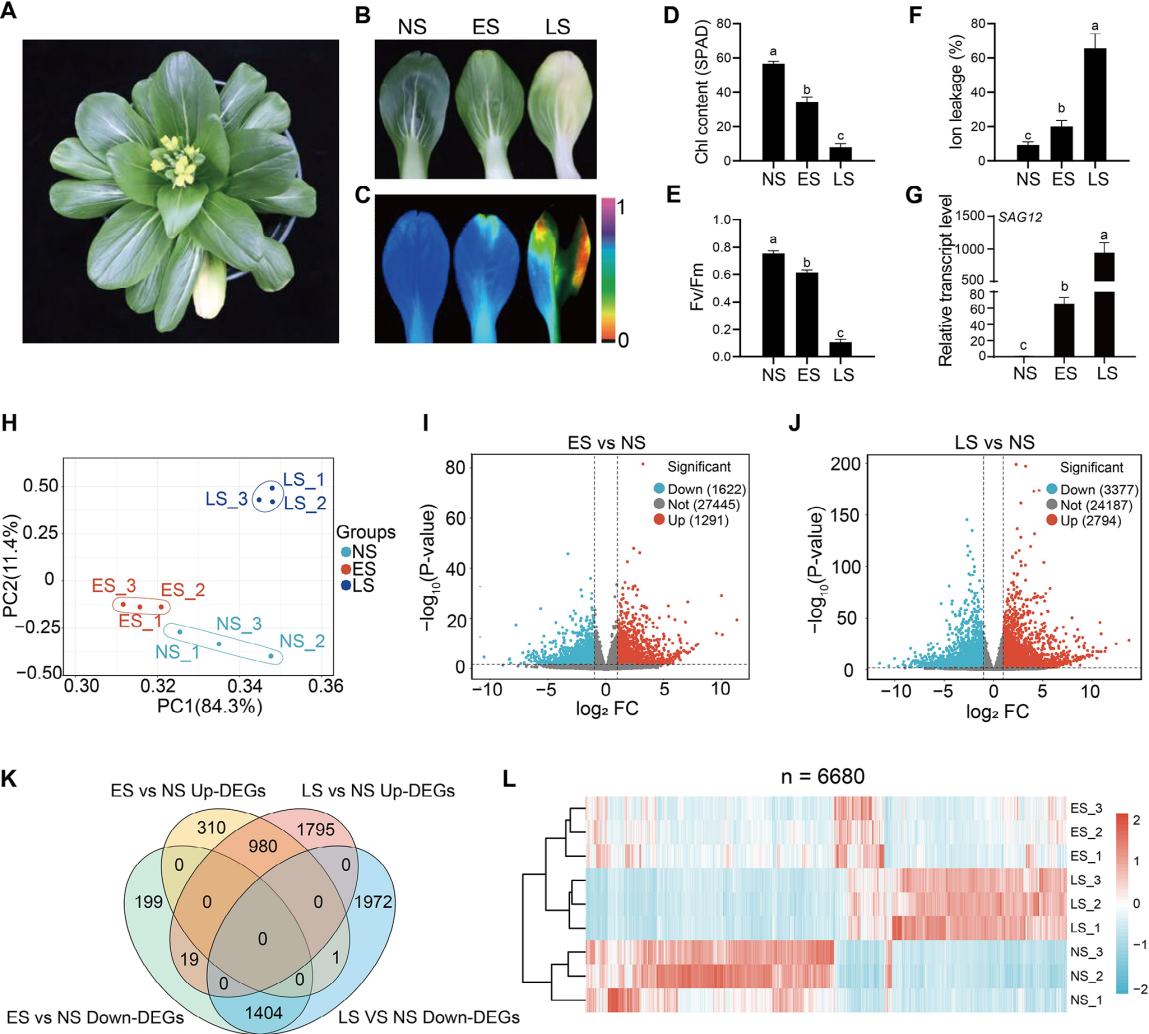
Phenotype and transcriptome analyses of senescent leaves in NHCC. A) Phenotype of an NHCC plant. Plants with one true leaf were vernalized at 4 °C for two weeks and then cultivated under normal growth conditions for 2 months before being photographed. B) Phenotypes of NHCC leaves at NS, ES, and LS stages. NS, ES, and LS denote non‐senescence, early‐senescence, and late‐senescence, respectively. C) Chlorophyll fluorescence imaging showing Fv/Fm values in NS, ES, and LS leaves. D−G) Chlorophyll content D), Fv/Fm ratio E), ion leakage percentage F), and relative transcript levels of *SAG12* G) in NS, ES, and LS leaves. Data are means ± SD (*n *= 3 biological replicates), different letters indicate significant differences (*p* < 0.05, one‐way ANOVA test). For each biological replicate, one leaf at each stage (NS, ES, and LS) was detached from a single plant, and then three same‐stage leaves from three plants were combined as one sample. H) PCA of the transcriptomes of the NS, ES, and LS leaves. I−J) Volcano plots of gene expression in ES versus NS I) and LS versus NS J). K) Venn diagram of DEGs between ES versus NS and LS versus NS comparisons. L) Heatmap showing DEGs identified in (K). DEGs were identified with a cut‐off threshold of |Log_2_Fold Change| ≥ 1 and *p*‐value < 0.01.

To investigate the molecular basis of leaf senescence in NHCC, we examined the leaf phenotypes of pakchoi, a major NHCC variety, under natural growth conditions (**Figure** **1**A). As natural leaf senescence initiates in an age‐dependent manner, we categorized three distinct stages for further analysis. Morphological changes showed that while the upper leaves remained nonsenescent (NS, no visible signs of yellowing), the middle and the bottom leaves had entered early senescence (ES, 30–40% chlorophyll was degraded), and late senescence (LS, >70% chlorophyll was degraded) stages, respectively (Figure 1B). Examination of chlorophyll content, maximum photochemical efficiency of PSII (Fv/Fm), ion leakage, and the expression of the senescence‐associated marker gene *SAG12* further confirmed a gradient progression of leaf senescence (Figure 1C–G).

To understand the transcriptome‐wide change in gene expression during leaf senescence in NHCC, we performed RNA‐seq analyses on NS, ES, and LS leaves. Principal component analysis (PCA) results showed that samples from different groups were separated along both axes (i.e., principal components), suggesting a significant transcriptome reprogramming during leaf senescence (Figure 1H). With a threshold setting at the absolute value of log_2_FoldChange ≥ 1 and *p*‐value < 0.01, we identified 2913 differentially expressed genes (DEGs) at the ES stage compared with the NS stage (Figure 1I), among which 1291 were up‐regulated and 1622 were down‐regulated. As expected, more DEGs were detected at the LS stage compared with the NS stage, with 2794 up‐regulated and 3377 down‐regulated genes (Figure 1J). A Venn diagram analysis revealed that 980 and 1404 genes were up‐regulated and down‐regulated at both stages relative to NS, respectively. In sharp contrast, only 20 genes showed an opposite expression change, i.e., up‐regulated at one stage but down‐regulated at the other (Figure 1K). A heatmap analysis of combined DEGs (*n* = 6680) from both the comparison groups (ES vs NS, LS vs NS) demonstrated the different dynamics of gene expression changes during leaf senescence (Figure 1L).

### 2.2 Identification of Senescence‐Associated Signaling Pathways in NHCC

**Figure 2:**
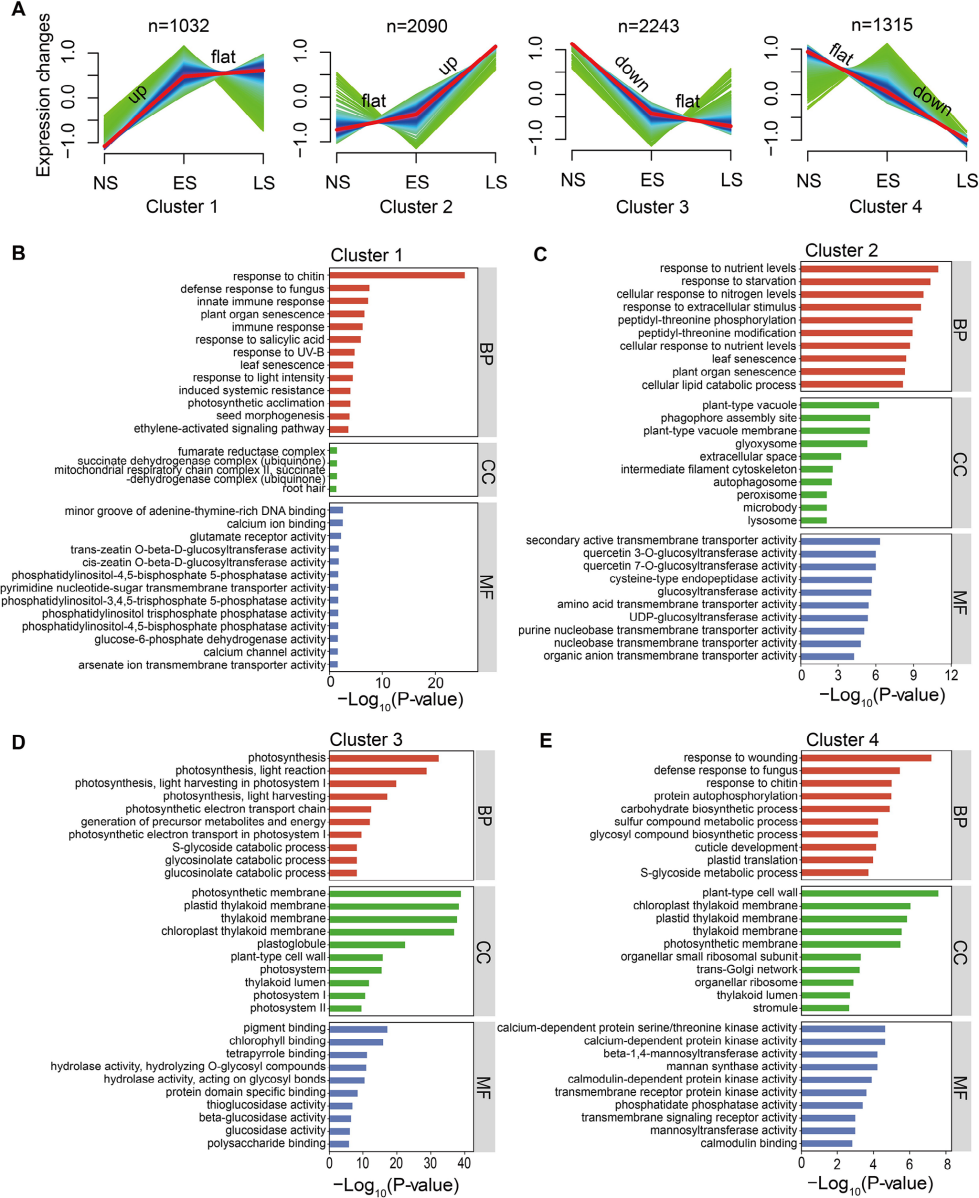
Pathway enrichment analysis of senescence‐associated DEGs in NHCC. A) Cluster analysis of 6680 senescence‐associated DEGs in NHCC. B) GO enrichment analysis of DEGs within cluster 1. C) GO enrichment analysis of DEGs within cluster 2. D) GO enrichment analysis of DEGs within cluster 3. E) GO enrichment analysis of DEGs within cluster 4.

The 6680 senescence‐associated DEGs were categorized into four major clusters using cluster analysis. Genes within cluster 1 and cluster 2 generally displayed an increasing trend in expression. Specifically, cluster 1 genes rapidly achieved high expression levels at the ES stage and sustained high levels throughout the LS stage, while cluster 2 genes were only marginally induced at the ES stage, but significantly upregulated at the LS stage. Conversely, genes in cluster 3 and cluster 4 exhibited a decreasing trend as leaf senescence progressed, with cluster 3 genes showing rapid down‐regulation at the ES stage and cluster 4 genes undergoing gradual down‐regulation throughout the senescence process (**Figure** **2**A).

Gene Ontology (GO) enrichment analysis indicated that cluster 1 genes were primarily associated with SA signaling and immune responses, followed by leaf and plant organ senescence and ET‐activated signaling (Figure 2B; Figure S1, Supporting Information). In addition to leaf and organ senescence pathways, cluster 2 genes were highly enriched in pathways responding to nutrient levels, starvation, and nitrogen levels, suggesting an active reprogramming and/or remobilization of nutrients at the LS stage (Figure 2C). The pathways enriched in cluster 3 were mainly associated with photosynthesis and glucosinolate/glycosinolate processes (Figure 2D). Intriguingly, the top enriched pathways in cluster 4 were significantly assigned to defenses against wounding and pathogens (Figure 2E). Based on these results, we inferred that both SA and ET signals are activated during the ES stage, accompanied by a decline in photosynthesis. As the leaf senescence progresses, organelles such as chloroplasts begin to break down, paralleled by the catabolism of macromolecules and the initiation of nutrient remobilization. It may also be envisioned that the down‐regulation of defense genes at the LS stage makes the leaves vulnerable to pathogen attack.

### 2.3 *BraC05g032350*/*EIN3A* and *BraC01g010480*/*CMT2* are Differentially Expressed during Leaf Senescence in NHCC

**Figure 3:**
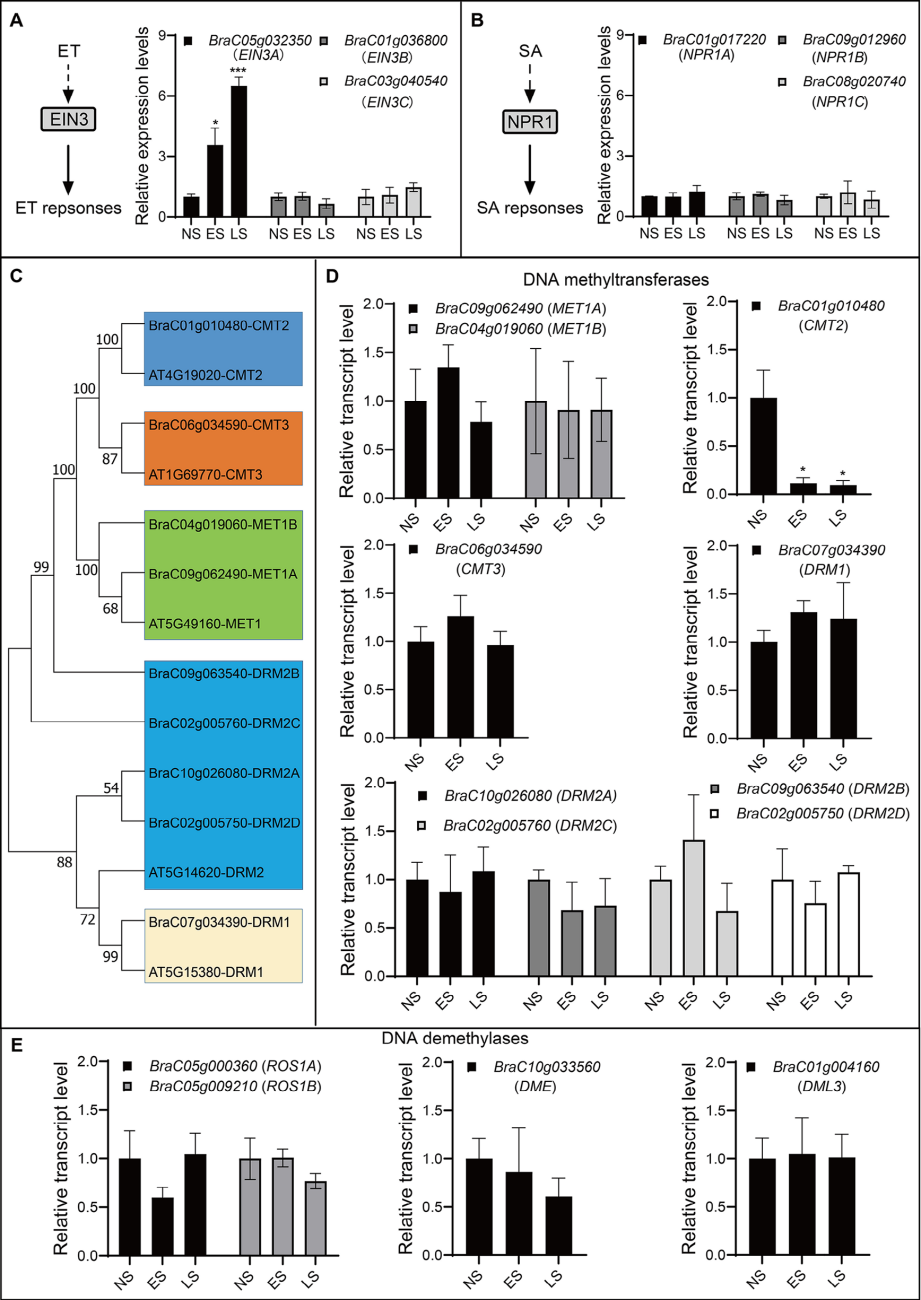
Expression patterns of phytohormone‐ and DNA methylation‐related genes during leaf senescence in NHCC. A) Relative transcript levels of *EIN3s* in senescent and non‐senescent leaves of NHCC. B) Relative transcript levels of *NPR1s* in senescent and non‐senescent leaves of NHCC. C) Phylogenetic tree of DNA methyltransferases from NHCC and *Arabidopsis thaliana*. D) Relative transcript levels of DNA methyltransferase genes in senescent and non‐senescent leaves. E) Relative transcript levels of DNA demethylase genes in senescent and non‐senescent leaves. Data are means ± SD (*n *= 3 biological replicates), ^*^ *p* < 0.05, ^***^ *p* < 0.001 (*t*‐test). Primer sequences are listed in Table S1 (Supporting Information).

Phytohormones play crucial roles in both the initiation and progression of leaf senescence.^[^ ^18^ ^]^ Previous studies revealed that ET and SA regulate leaf senescence in an EIN3‐ and NPR1‐dependent manner, respectively.^[^ ^8^, ^19^ ^]^ As ET and SA signals were activated at the ES stage in NHCC (Figure 2B), we further checked the expression profiles of *EIN3* and *NPR1* during leaf senescence. The NHCC genome encodes three *EIN3*s (*BraC05g032350*/*EIN3A*, *BraC01g036800*/*EIN3B*, *BraC03g040540*/*EIN3C*) and three *NPR1s* (*BraC01g017220*/*NPR1A*, *BraC09g012960*/*NPR1B*, *BraC08g020740*/*NPR1C*).^[^ ^16a^ ^]^ qPCR results showed that only *EIN3A* was significantly up‐regulated during leaf senescence (**Figure** **3**A,B), suggesting a functional analogy to Arabidopsis *EIN3* in regulating leaf senescence. Gene transcription is regulated by both trans‐acting regulators and cis‐acting epigenetic status, including DNA methylation and histone modifications.^[^ ^20^ ^]^ In this study, we focused on the expression profiles of genes that encode DNA methylation and demethylation enzymes. The NHCC genome contains nine DNA methyltransferase genes (Figure 3C) and four DNA demethylase genes. All tested genes showed no significant changes during leaf senescence except for *CMT2*, which was dramatically down‐regulated at both the ES and LS stages (Figure 3D,E; Figure S2, Supporting Information), implying that CMT2‐mediated DNA methylation may participate in regulating leaf senescence in NHCC.

### 2.4 EIN3A Directly Represses the Expression of *CMT2* to Downregulate CHH Methylation During Leaf Senescence

**Figure 4:**
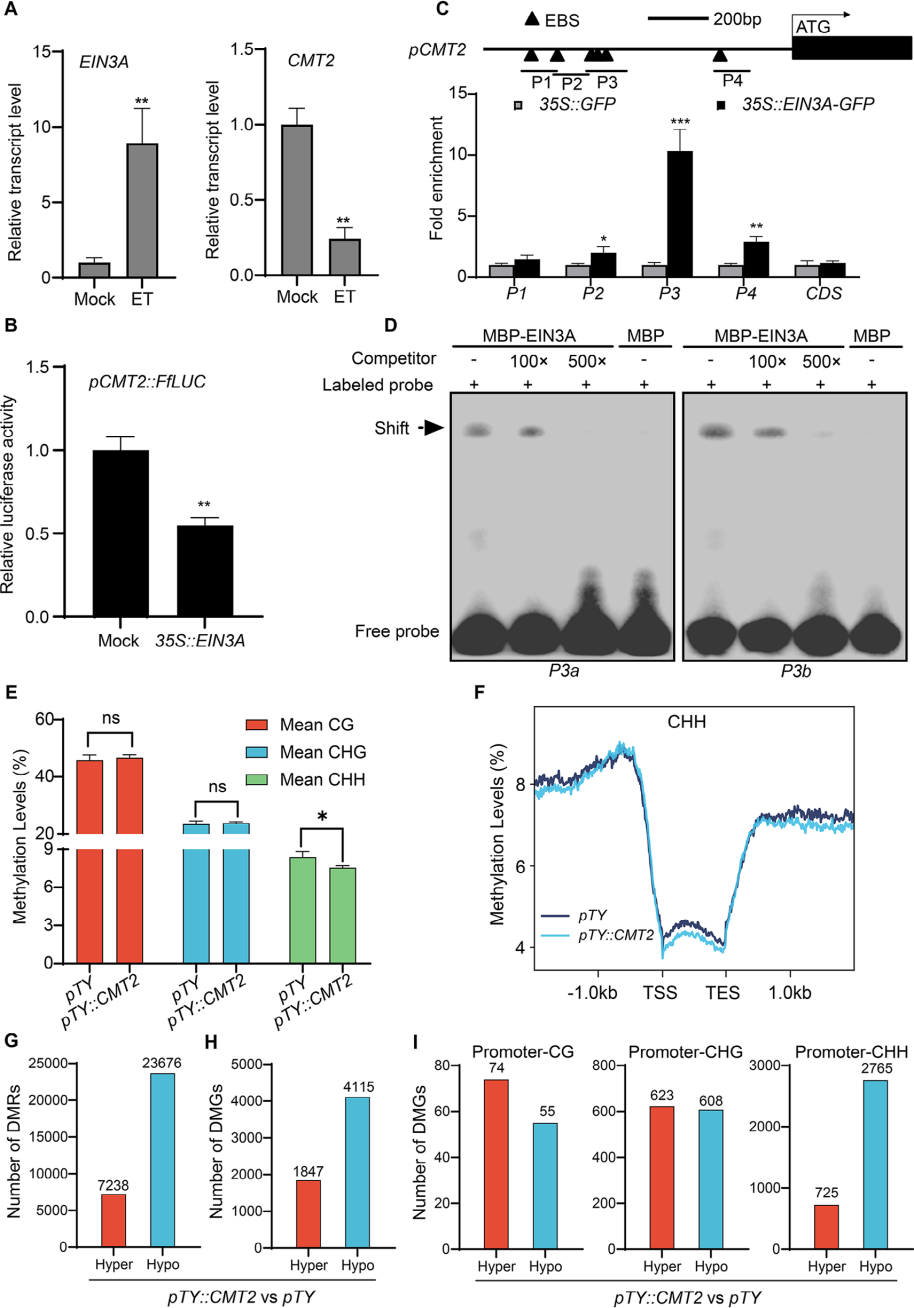
EIN3A inhibits the promoter activity of *CMT2*. A) Relative expression levels of *EIN3A* and *CMT2* in ET‐ and mock‐treated NHCC leaves. Detached leaves were used for treatment, and gene expression was examined after a 3‐day treatment. The fourth true leaf of one‐month‐old NHCC plants was used for this experiment. B) Dual‐luciferase analysis of the effect of EIN3A on the promoter activity of *CMT2*. *pCMT2*::*FfLUC* and *p35S::EIN3A* were co‐expressed in tobacco leaves via the mediation of *Agrobacterium tumefaciens*, with an empty vector being used as a mock treatment. C) ChIP‐qPCR analysis of EIN3A binding to the *CMT2* promoter. *p35S::EIN3A‐GFP* and *p35S::GFP* were transfected into the cotyledons of two‐week‐old NHCC plants. ChIP was conducted two days post‐trasfection. Fragment enrichments were normalized to *ACTIN2*, with CDS of *CMT2* being used as an internal control. Data are means ± SD (*n *= 3 biological replicates), ^*^ *p* < 0.05, ^**^ *p* < 0.01, ^***^ *p* < 0.001 (*t*‐test). Primer sequences are listed in Table S1 (Supporting Information). D) EMSA analysis of the interaction of EIN3A and EBSs located on *CMT2* promoter. Biotin‐labeled fragments were used as probes, and unlabeled fragments were used as competitors. E) Genome‐wide methylation levels of CG, CHG, and CHH contexts in *pTY* and *pTY::CMT2* plants. One‐month‐old NHCC plants were subjected to VIGS, and samples were collected for WGBS five days post VIGS. F) Average CHH methylation levels across genes and their flanking regions in *pTY* and *pTY::CMT2* plants. G,H) DMRs G) and DMGs H) numbers in *pTY::CMT2* plants relative to *pTY* plants. I) Number of hyper‐ and hypo‐DMGs with promoter generating DMRs in *pTY::CMT2* plants versus *pTY* plants.

Given that ET is a major phytohormone in activating leaf senescence, we examined the effect of ET and EIN3A on the expression of *CMT2*. As shown in **Figure** **4**A, *EIN3A* was significantly upregulated following a 3‐day ET treatment, while *CMT2* was significantly reduced. To determine whether EIN3A directly regulates *CMT2*, we performed a dual‐luciferase assay. For functional comparison, we included two *ORE1* homologs (*BraC04g014200* and *BraC07g09800*), known targets of EIN3 in Arabidopsis. Although transient over‐expression of EIN3A activated the promoters of both *ORE1* homologs, it strongly suppressed the *CMT2* promoter (Figure S3, Supporting Information; Figure 4B). We then carried out a motif screening over the promoter of *CMT2* and identified six putative EIN3 binding sites (EBSs) on four distinct regions (P1 to P4) (Figure 4C). Chromatin immunoprecipitation‐qPCR (ChIP‐qPCR) analysis revealed a significant enrichment of EIN3A on the P2, P3 and P4 regions, but not on the distal P1 region. The P3 region, harboring three tandem EBSs, showed the strongest EIN3A binding (Figure 4C). We next conducted an electrophoretic mobility shift assay (EMSA) to test whether EIN3A could directly bind to the P3 region in vitro. As shown in Figure 4D, incubating EIN3A‐MBP, but not MBP, with P3a or P3b probes (P3a and P3b contain the first two EBSs and the last EBS within P3 region, respectively), resulted in shifted bands, whereas excessive addition of unlabeled competitor oligos abolished the band shift (Figure 4D). These results suggest that ET suppresses the expression of *CMT2* through a direct inhibition of EIN3A to the promoter activity of *CMT2*.

To assess whether CMT2 and EIN3A impact DNA methylation in vivo, we performed virus‐induced gene silencing (VIGS) targeting *CMT2* or *EIN3A* and conducted whole‐genome bisulfite sequencing (WGBS). *CMT2* silencing (*pTY::CMT2*) significantly reduced DNA methylation levels in the CHH context, but not CG and CHG contexts (Figure 4E). By contrast, *EIN3A* silencing (*pTY::EIN3A*) barely affects global DNA methylation (Figure S4, Supporting Information). Further analysis revealed that CHH methylation was reduced across gene bodies and flanking regions in *CMT2*‐silencing plants (Figure 4F). In accordance with this, differential methylation analysis identified more hypo‐methylated regions (hypo‐DMRs) and genes (hypo‐DMGs) than hyper‐methylated ones in *CMT2*‐silenced plants (Figure 4G,H). Analysis of promoter methylation levels further supported that CMT2 mainly contributed to CHH methylation (Figure 4I).

### 2.5 Reduced DNA Methylation Accelerates Leaf Senescence in NHCC

**Figure 5:**
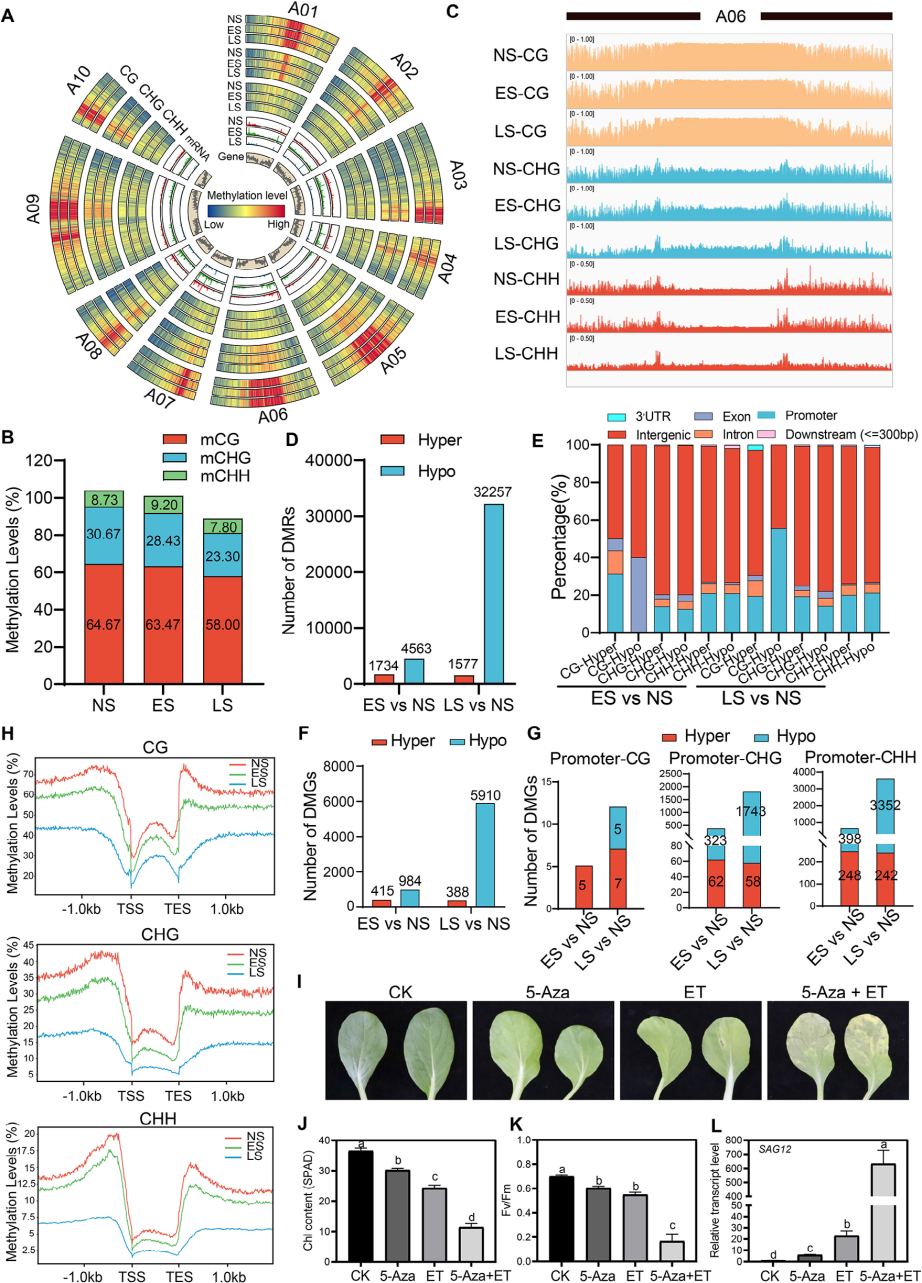
DNA methylation inhibits leaf senescence in NHCC. A) Circos plot showing the gene densities, mRNA, and DNA methylation levels of CG, CHG, and CHH contexts in the NS, ES, and LS leaf samples. B) Genome‐wide methylation levels of CG, CHG, and CHH contexts in the NS, ES, and LS leaf samples. C) IGV browser showing the DNA methylation level of the A06 chromosome in the NS, ES, and LS leaf samples. D) Numbers of DMRs in the ES and LS leaf samples in comparison with the NS leaf sample. E) Percentages of DMRs located in different genomic regions in the ES and LS leaf samples in comparison with the NS leaf sample. F) Numbers of DMGs in the ES and LS leaf samples in comparison with the NS leaf sample. G) Numbers of DMGs located in promoter regions in the ES and LS leaf samples in comparison with the NS leaf sample. H) Average DNA methylation level across genes and their flanking regions. I) Phenotypes of NHCC leaves after chemical treatments. Photographs were taken 5 days post‐treatments. The fourth true leaf of one‐month‐old NHCC plants was used for this experiment. J−L) Chlorophyll contents J), Fv/Fm ratios (K), and relative transcript levels of *SAG12* L) in the chemical‐treated NHCC leaf samples. Data are means ± SD (*n *= 3 biological replicates), and different letters indicate significant differences at *p* < 0.05 (one‐way ANOVA test). Primer sequences are listed in Table S1 (Supporting Information).

To explore the role of DNA methylation in leaf senescence, we conducted WGBS on NS, ES, and LS leaf samples (**Figure** **5**A). Generally, global DNA methylation levels showed a gradual descending trend during leaf senescence (Figure 5B,C; Figure S5, Supporting Information). An analysis of DMRs revealed significantly more hypo‐DMRs (4563 at the ES stage and 32257 at the LS stage) than hyper‐DMRs (1734 at ES and 1577 at LS) as compared to the NS stage (Figure 5D). These DMRs were largely located in the intergenic and promoter regions, followed by introns and exons (Figure 5E). To investigate the impact of DMRs on gene expression, we focused on DMRs that mapped to gene bodies and promoter regions (1 kb). In total, 984 hypo‐DMGs and 415 hyper‐DMGs were detected at the ES stage, and 5910 hypo‐DMGs and 388 hyper‐DMGs were detected at the LS stage (Figure 5F). Given the significant connection between promoter DNA methylation and gene expression, we further counted DMGs that occurred in promoter regions. Despite the highest abundance of CG methylation at the global level (Figure 5B), promoter DMGs mainly occurred in the CHG and CHH contexts (Figure 5G). On a gene‐centered view, all three contexts (i.e., CG, CHG, and CHH) showed declined trends during the progression of leaf senescence, with the most dramatic decline occurring in the 1 kb regions upstream of transcription start sites (TSS) and downstream of transcription end sites (TES) (Figure 5H).

To investigate the crosstalk between DNA demethylation and ET‐induced leaf senescence, we treated detached NHCC leaves with a broad‐spectrum DNA methylation inhibitor (5‐Azacytidine, 5‐Aza) and/or ET, and leaf senescence syndromes (i.e., Chl content, Fv/Fm, and *SAG12* expression) were monitored at 5 days post‐treatment. Similar to ET, 5‐Aza treatment also efficiently promoted leaf senescence. Intriguingly, a combined treatment with both ET and 5‐Aza caused a more severe leaf senescence syndrome than either single treatment (Figure 5I–L). These findings suggest that DNA methylation may serve as a “braking” mechanism in both ET‐ and age‐triggered leaf senescence in NHCC.

### 2.6 CMT2 and EIN3A Mediate ET‐ and Age‐Triggered Leaf Senescence in NHCC

**Figure 6:**
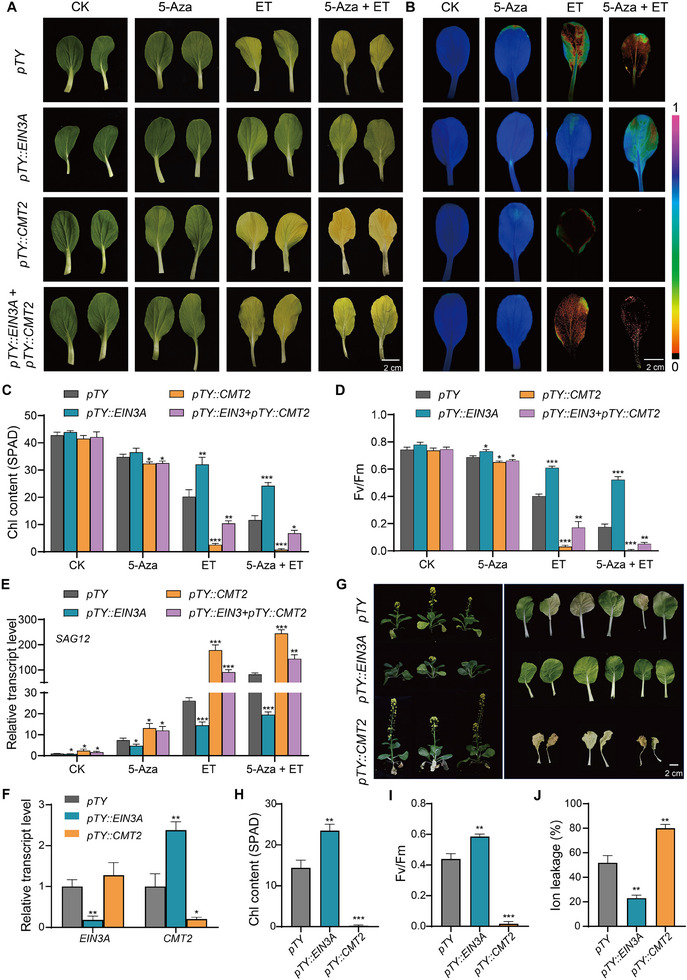
CMT2 and EIN3A regulate leaf senescence in NHCC. A) Phenotypes of *CMT2*‐ and *EIN3A*‐silenced NHCC leaves after treatments. The chemical treatments were carried out on detached true leaves, with H_2_O treatment being used as a control. Photographs were taken five days post‐treatments. The fourth true leaf of one‐month‐old NHCC plants was used for this experiment. B) Chlorophyll fluorescence imaging showing Fv/Fm values in *CMT2*‐ and *EIN3A*‐silenced leaves after treatments. C−E) Chlorophyll content C), Fv/Fm ratio D) and relative transcript levels of *SAG12* E) in *CMT2*‐ and *EIN3A*‐silenced leaves after treatments. *pTY* transfected leaves were used as a negative control. F) Examination of VIGS efficacy. qPCR analysis was conducted using cotyledons after VIGS. G) Phenotypes of *CMT2*‐ and *EIN3A*‐silenced caixin plants and their first and second true leaves after 50‐day growth. H−J) Chlorophyll content H), Fv/Fm ratio I), and ion leakage percentage J) in the first and second true leaves of *CMT2*‐ and *EIN3A*‐silenced plants after 50‐day growth. Data are means ± SD (*n *= 3 biological replicates), ^*^ *p* < 0.05, ^**^ *p* < 0.01, ^***^ *p* < 0.001 (*t*‐test). Primer sequences are listed in Table S1 (Supporting Information).

We silenced *CMT2* and *EIN3A* in NHCC using VIGS system, and the detached leaves from *pTY:CMT2‐*, *pTY:EIN3A‐*, and *pTY*‐transfected plants were then subjected to 5‐Aza and ET treatments. As compared with *pTY* leaves, *CMT2* silencing slightly accelerated 5‐Aza‐induced leaf senescence but strongly promoted ET‐triggered leaf senescence. In sharp contrast, *EIN3A* silencing substantially, but not completely, attenuated ET‐triggered leaf senescence. Interestingly, co‐silencing of *EIN3A* and *CMT2* produced intermediate senescence phenotypes compared to single silencing. The incomplete suppression of leaf senescence could be due to residual EIN3A activities after VIGS or functional redundancy conferred by EIN3B or unknown transcription factors. Collectively, these results suggest that reduced DNA methylation (via 5‐Aza treatment or *CMT2* silencing) accelerates ET‐triggered leaf senescence through mechanisms at least partially dependent on EIN3A (**Figure** **6**A–E; Figure S6, Supporting Information).

We further analyzed the role of CMT2 and EIN3A on natural leaf senescence in a fast bolting NHCC variety caixin.^[^ ^16b^ ^]^ As shown in Figure 6F, the expression levels of *EIN3A* and *CMT2* were specfically suppressed in *pTY::EIN3A* and *pTY::CMT2* plants, suggesting VIGS was effective. Consistent with the results in pakchoi, we observed upregulated *CMT2* expression when EIN3A was silenced. We then monitored leaf senescence syndromes of *CMT2*‐ and *EIN3A*‐silenced plants under natural growth conditions. Compared with the *pTY* plants, *CMT2* silencing significantly accelerated the age‐triggered leaf senescence, while the *EIN3A* silencing showed remarkably delayed leaf senescence (Figure 6G–J; Figure S7, Supporting Information). Together, these findings suggest that CMT2 and EIN3A antagonistically regulate ET‐ and age‐triggered leaf senescence in NHCC.

### 2.7 CMT2‐Mediated DNA Methylation Inhibits EIN3A‐Mediated *SAG* Induction

**Figure 7:**
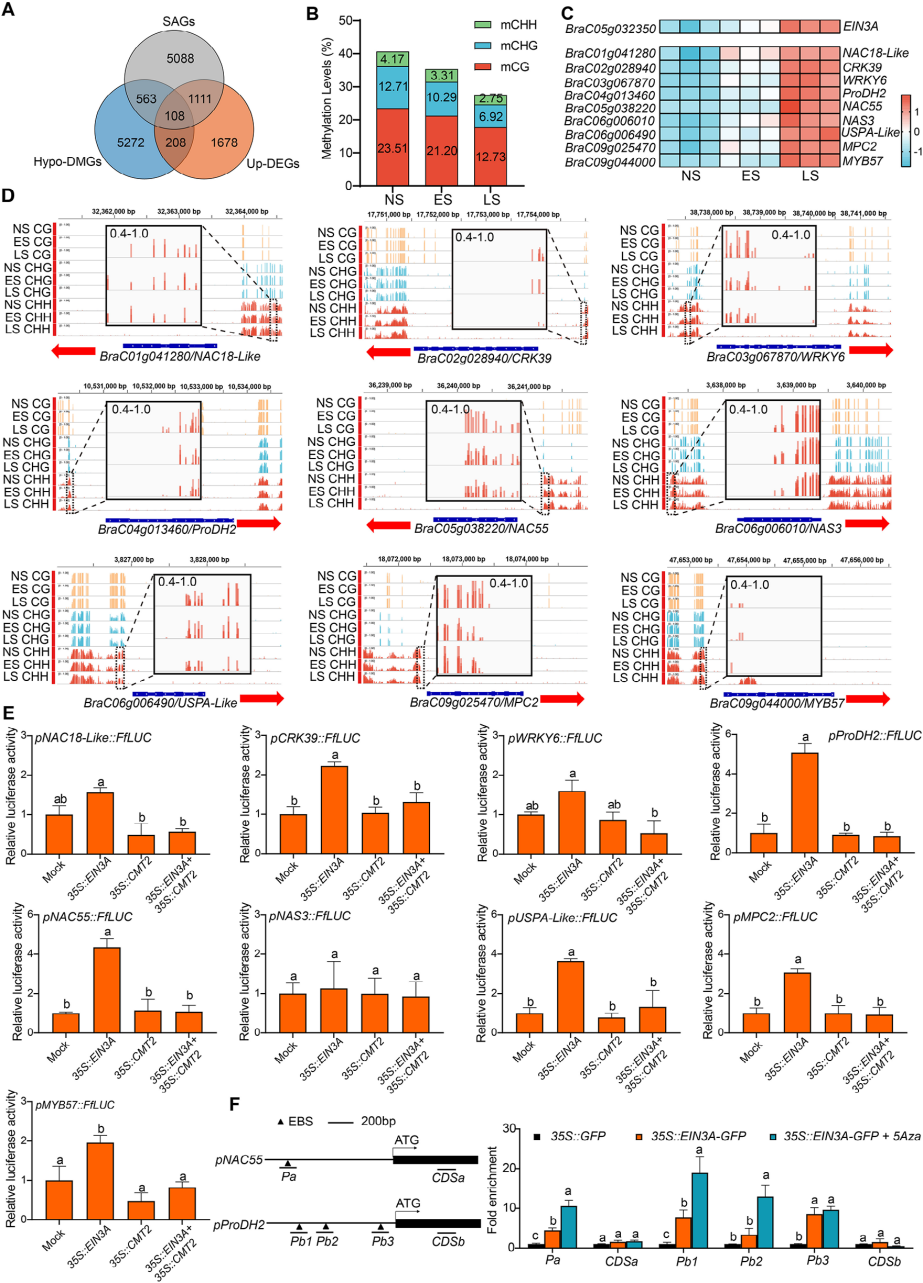
EIN3A and CMT2 antagonistically regulate *SAGs’* expression. A) A Venn diagram showing the overlap of *SAGs*, upregulated *DEGs*, and hypo‐*DMGs*. B) Methylation levels of upregulated and hypo‐methylated *SAGs* in CG, CHG, and CHH contexts. C) A heatmap of *EIN3A* and nine *SAGs* expression profiles during leaf senescence in NHCC. D) IGV displays the methylation levels of CG, CHG, and CHH contexts on nine *SAGs’* promoters. DMRs were shown in the amplified frames. E) Dual‐luciferase analyses of the effects of EIN3A and CMT2 on the activity of the nine *SAGs’* promoters. *pSAGs*::*FfLUC* were co‐introduced into tobacco with *p35S::EIN3A*, or *p35S::CMT2*, or both of them via the mediation of *Agrobacterium tumefaciens*, with empty vector‐transfected samples being used as the controls. F) ChIP‐qPCR assay of the interaction of EIN3A and *SAGs’* promoters. *p35S::EIN3A‐GFP* and *p35S::GFP* were transfected into the cotyledons of two‐week‐old NHCC plants. 5‐Aza was sprayed evenly on the surface of cotyledons, and ChIP was conducted 2 days post inoculation of *Agrobacterium tumefaciens*. Fragment enrichments were normalized to *ACTIN2*, with CDS of *NAC55* and *ProDH2* being used as an internal control. Data are means ± SD (*n *= 3 biological replicates), and different letters indicate significant differences at *p* < 0.05 (one‐way ANOVA test). Primer sequences are listed in Table S1 (Supporting Information).

To probe the relationship between declined DNA methylation and induced *SAG* expression, we analyzed the associations of hypo‐DMGs, upregulated DEGs, and annotated *SAGs* (Figure S8, Supporting Information). A Venn diagram showed that a total of 108 *SAGs* were significantly up‐regulated and hypo‐methylated in senescent leaves (**Figure** **7**A). Analyzing the methylation status of these 108 genes revealed that all CG, CHG, and CHH contexts were reduced during leaf senescence (Figure 7B). We further examined the expression patterns of these 108 genes and identified nine *SAGs* (i.e., *NAC18‐Like*, *CRK39*, *WRKY6*, *ProDH2*, *NAC55*, *NAS3*, *USPA‐Like*, *MPC2*, and *MYB57*) showing a similar expression trend to *EIN3A*, and importantly, the promoters of these nine genes showed reduced CHH methylation levels, albeit to varying degrees, during leaf senescence (Figure 7C,D). We surmised that some of these *SAGs* may be targets of EIN3A, and DNA modification on their promoters hinders EIN3A binding. To test this hypothesis, we examined the effects of EIN3A and CMT2 on the promoter activity of these nine *SAGs*. As shown in Figure 7E, overexpression of EIN3A significantly activated the promoter activity of six out of nine *SAGs*, with the *NAC55* and *ProDH2* promoters showing the most prominent induction. In contrast, CMT2 alone did not exert an obvious effect on these promoters. Strikingly, overexpression of CMT2 antagonized EIN3A's activation on the *SAG* promoters (Figure 7E). To investigate whether DNA methylation influences EIN3A‐mediated *SAG* induction, we conducted ChIP‐qPCR to assess the binding of EIN3A on the promoter regions of *NAC55* and *ProDH2* with or without 5‐Aza treatment. The results showed that EIN3A is significantly bound to the EBS motifs within *ProDH2* and *NAC55* promoters in vivo. After the 5‐Aza treatment, the occupancy of EIN3A was further enhanced at three out of four EBS fragments (Figure 7F). Taken together, these results suggest that DNA methylation on *SAG* promoters may interfere with the binding of EIN3A, thereby impairing their induction during leaf senescence.

## 3 Discussion

The initiation and progression of leaf senescence are modulated by a combined effect of endogenous signals and external stimuli.^[^ ^1a^ ^]^ Among these, phytohormones, including ET, jasmonic acid (JA), abscisic acid (ABA), and SA positively regulate leaf senescence.^[^ ^1b,c^ ^]^ In this study, we detected significant GO enrichment of ET and SA signaling pathways, but not other phytohormone pathways, at the early stage of leaf senescence in NHCC (Figure 2). Previous studies have shown that EIN3 and NPR1 serve as the core hubs of ET and SA signaling pathways, respectively, in regulating leaf senescence.^[^ ^8^, ^19^, ^21^ ^]^ Intriguingly, we only detected an induction of *EIN3A* during leaf senescence in NHCC, with no significant changes in the expression of *EIN3B*, *EIN3C*, and three *NPR1* homologous genes (Figure 3A,B). EIN3 has been shown to interact with NPR1 to mediate the synergistic regulation of ET and SA on leaf senescence.^[^ ^9^ ^]^ Ubiquitination and phosphorylation are shown to regulate the transcriptional activity and stability of NPR1 during systemic acquired resistance (SAR).^[^ ^22^ ^]^ Therefore, it is possible that NPR1 is activated post‐translationally during leaf senescence. Consistent with this, the immune response signal was robustly activated in senescent leaves. These observations also imply an associative regulation of ET and SA on plant senescence and immunity (Figure 2).^[^ ^19^, ^21^, ^23^ ^]^

DNA methylation plays an important role in regulating plant development and stress responses.^[^ ^24^ ^]^ A large body of studies have demonstrated that DNA methylation level correlates with age in humans and other mammals, leading to the establishment of epigenetic clock theory.^[^ ^25^ ^]^ In plants, active demethylation is linked to fruit ripening in tomato and strawberry,^[^ ^14^, ^15^ ^]^ while other studies have reported DNA hypermethylation during fruit ripening in orange and pear.^[^ ^26^ ^]^ These findings suggest that the regulatory relationship between DNA methylation and organ ripening/senescence could be species‐specific. In *Arabidopsis thaliana*, DNA demethylase *DML3* is gradually up‐regulated during leaf senescence and its dysfunction leads to hypermethylation of *SAGs*’ promoters.^[^ ^14^ ^]^ In the present study, we observed a gradual decline in genome‐wide DNA methylation levels during leaf senescence in NHCC, yet there were no significant changes in the expression of DNA demethylase genes (Figures 3E and 5). Notably, *CMT2* was markedly downregulated in the senescent leaves and *CMT2* silencing significantly reduced CHH methylation (Figures 3D and 4E–I). These observations suggest that, although DNA methylation levels declines during leaf senescence in both *Arabidopsis thaliana* and NHCC, the mechanisms underlying this process differ between the two species. We propose that the CMT2‐mediated maintenance of methylation plays a “braking” role during leaf senescence in NHCC, which significantly differs from the previous findings, i.e., downregulation of *de novo* DNA methylation or activation of DNA demethylation in initiating plant organ ripening and senescence in other plant species.^[^ ^13^ ^]^

**Figure 8:**
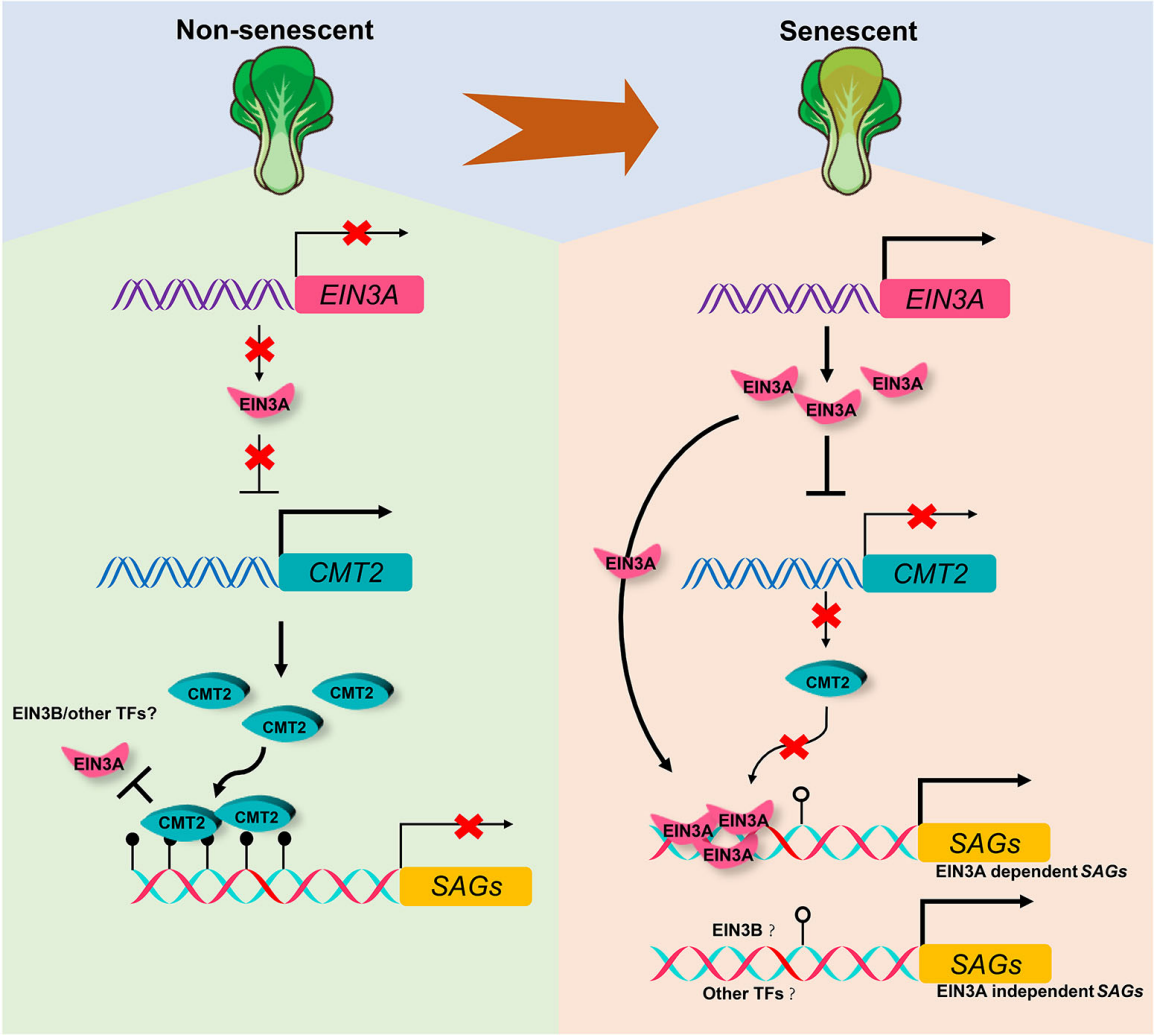
A proposed model of EIN3A and CMT2 antagonistically regulating leaf senescence in NHCC.

In eukaryotes, transcription factors usually coordinate with epigenetic regulators to regulate gene expression.^[^ ^27^ ^]^ However, the initial signal that triggers plant leaf senescence and fruit ripening remains an enigma.^[^ ^13^, ^28^ ^]^ We found that the accumulation of *EIN3A* transcripts precedes the downregulation of *CMT2* (Figure S9, Supporting Information). More importantly, EIN3A directly targets and suppresses the expression of *CMT2* (Figure 4). Conversely, the reduction of CMT2‐dependent DNA methylation subsequently releases EIN3A's activation on *SAGs* (Figure 7). Although ET plays a broad regulatory role in plant development, it initiates senescence only in leaves that have reached a certain age, a phenomenon known as the “senescence window.”^[^ ^29^ ^]^ The mechanisms underlying this specificity are barely understood. Our findings suggest that DNA methylation on the promoters of *SAGs* may serve as a barrier to EIN3A's functionality, potentially preventing premature induction of *SAGs*. As plants age, DNA methylation gradually declines, enabling EIN3A, and likely other senescence‐associated transcription factors, to bind to certain *SAGs* and initiate leaf senescence (Figures 7 and **8**). Interestingly, we didn't observe a direct interaction between EIN3A and the DNA methyltransferase CMT2, but the reduction of DNA methylation increased EIN3A's binding affinity to its targets (Figure S10, Supporting Information; Figure 7). These findings suggest that DNA methylation may counteract EIN3A's activation on *SAGs*, ensuring that leaves do not senesce prematurely. Whether this regulatory mechanism is conserved in other plants awaits to be investigated.

## 4 Conclusion

Maintenance of DNA methylation influences leaf senescence, which involves CMT2‐mediated DNA methylation at euchromatin regions in NHCC, and this mechanism significantly differs from the previous findings in other species. We also identified the EIN3A‐CMT2 module mediating the antagonism of DNA methylation and ET signal in regulating leaf senescence in NHCC (Figure 8). These findings not only unravel a regulatory module of leaf senescence in NHCC, but also contribute to the elucidation of the relationship between ET signaling and DNA methylation during plant growth and development.

In non‐senescent NHCC, CMT2 mediates the maintenance of DNA methylation on the promoters of *SAGs*, thereby inhibiting EIN3A untimely activating *SAGs*’ expression. Along with the development of NHCC plants, an aging‐impelled striking upregulation of *EIN3A* strongly suppresses the expression of *CMT2* to reduce the methylation level, which releases the activation of EIN3A on *SAGs*, thus initiating and accelerating the progression of leaf senescence in NHCC.

## 5 Experimental Section

### Plant Material and Growing Conditions

Two NHCC varieties, namely *Brassica rapa* ssp*. chinensis* var. *communis* (pakchoi) and *Brassica rapa* ssp*. chinensis* var*. parachinensis* (caixin) were used in the present study. Pakchoi was used in all experiments except those in Figure 6G–J and Figure S7 (Supporting Information), in which caixin was used. NHCC was cultivated at 22 °C under long‐day conditions (16‐h light/8‐h dark) with light intensity at ≈200 µmol m^−2^ s^−1^ and humidity at 60%. For analyzing the natural growth and development of pakchoi, one‐true‐leaf stage plants were vernalized at 4 °C for 2 weeks and transferred to 22 °C for further growth.

### Measurement of Chlorophyll Content

Chlorophyll content was determined using a SPAD‐502 Plus chlorophyll meter (Konica Minolta, Japan).^[^ ^19^ ^]^

### Measurement of Fv/F_M_ Ratio

The maximum photochemical efficiency of PSII (Fv/Fm) was determined by a chlorophyll fluorescence imaging system (MAXI‐IMAGING‐PAM, Heinz‐Walz, Germany) according to the manufacturer's instructions.

### Measurement of Ion Leakage

Measurement of ion leakage was conducted as described previously with some minor modifications.^[^ ^21^ ^]^ Briefly, to be detected samples were placed in a centrifuge tube with deionized water immersed in it. After a 2‐h shake in 25 °C shaker, the conductivity value of the solution was measured by a digital conductivity meter (Waterproof ECTestr11+ Multi‐Range Tester), and the detected value was recorded as T1. Then, the detected sample was further boiled for 30 min, and the secondary value of the solution was measured and recorded as T2. Ion leakage was calculated as a ratio of T1 and T2.

### Chemical Treatments on NHCC

For the treatment of living plants, chemicals were sprayed gently on the surface of plants via a spray bottle. For treatment of detached leaves, leaves were placed on the filter paper soaked with indicated chemical solution. The final working concentration of 5‐azacytidine is 500 µm. ET treatment was conducted in a sealed glass desiccator with leaves placed on the filter paper soaked with H_2_O. ET was produced via a chemical reaction via mixing 5 mm Na_2_HPO_3_ and 0.5 m ethephon, the final concentration of ET was controlled at ≈100 µL L^−1^.

### RNA Extraction and qPCR

RNA was extracted using RNAiso PLUS (Takara), and cDNA synthesis was generated using a PrimeScript RT Master Mix kit (Takara). mRNA level was measured using a TB Green Premix Ex Taq II (Tli RNaseH Plus) kit (Takara). Quantitative real‐time PCR (qPCR) was conducted on the CFX Connect Real‐Time PCR Detection System (Bio‐Rad), and the reaction procedure was set as follows: 95 °C for 2 min, 40 cycles of 95 °C for 15 s, 56 °C for 30 s, and then 72 °C for 30 s. Primers for qPCR are listed in Table S1 (Supporting Information).

### RNA Sequence and Data Analysis

Samples were harvested into liquid nitrogen and stored in −80 °C fridge for further analysis. RNA extraction and library construction were entrusted by Neo‐bio technology company (Shanghai), and RNA sequencing was conducted on a NovaSeq 6000 Sequencing System. The obtained clean reads were mapped to *Brassica rapa* NHCC001 genome using the hisat2 program.^[^ ^16a^ ^]^ RSEM software was used to read annotation and FPKM calculation.^[^ ^30^ ^]^ Differential expression analysis was performed using DESeq2, and DEGs were defined with the absolute value of log_2_ Fold Change ≥ 1 and *p*‐value < 0.01.^[^ ^31^ ^]^ GO and KEGG enrichment analysis was displayed by using R package ggplot2, cluster analysis of gene expression was carried out by using R package Mfuzz. Venn diagram and heatmap were presented by using TBtools.^[^ ^32^ ^]^ The raw sequencing data were deposited to NCBI Sequence Read Archive (SRA) in BioProject under accession numbers PRJNA1031375.

### Whole‐genome Bisulfite Sequencing and Data Analysis

Genomic DNA extraction and whole‐genome bisulfite sequencing were conducted on the NovaSeq 6000 sequencing platform entrusted by Neo‐bio technology company (Shanghai). Reads were mapped to *Brassica rapa* NHCC001 genome using Bismark software and allowing for a maximum of one mismatch.^[^ ^33^ ^]^ The Bismark methylation extractor was employed to call methylated cytosines from uniquely mapped reads. Methylation level was estimated using cytosines with at least four reads and calculated by dividing the number of methylated C by the sum of C and T. The lambda sequence was utilized to calculate the bisulfite conversion rate.

To identify differentially methylated regions (DMRs), the R package DMRcaller was utilized to divide the entire genome into bins of equal length and calculate the average methylation level of each bin, employing the bins algorithm with the following parameters: method = “bins,” binSize = 100, min Reads PerCytosine = 4, *p* Value Threshold = 0.01, and the absolute methylation difference for CG, CHG, and CHH were set at 0.4, 0.2, and 0.1, respectively.^[^ ^34^ ^]^ Sequence regions overlapping with the DMRs in the gene body or the promoter region (1 kb upstream of the transcription start site) were identified as differentially methylated genes (DMGs). Circos plot of DNA methylation level was presented by TBtools.^[^ ^32^ ^]^ IGV browser was employed to visualize the methylation peak map. The raw sequencing data were deposited to NCBI SRA in BioProject under accession numbers PRJNA1035634.

### Dual Luciferase Reporter Assay

The tested promoters were cloned into *pGreenII 0800‐LUC* plasmid, and effectors, i.e., *EIN3A* and *CMT2* were cloned into *pCHF3* plasmid for overexpression. *pGreenII 0800‐LUC* harboring promoters and *pCHF3* harboring effectors were respectively introduced into *Agrobacterium tumefaciens* strain *GV3101‐pSoup‐p19* (Weidi). After a 2‐h activation under light, different plasmid‐introduced *Agrobacterium tumefaciens* cells were mixed and infiltrated into the 4‐true‐leaf *Nicotiana benthamiana* leaves. After overnight cultivation, *Agrobacterium tumefaciens*‐infiltrated tissues were harvested for examination of Firefly and Renilla luciferase activities with the help of a TransDetect Double‐Luciferase Reporter Assay Kit (TransGen Biotech). Fluorescence was monitored with a Synergy two multi‐mode microplate reader (Bio‐Tek) according to the manufacturer's instructions.

### Transient Gene Expression in NHCC Cotyledons

The coding sequence (CDS) without termination codon of indicated gene was inserted into *pCAMBIA1306* plasmid harboring a GFP tag. Transient gene overexpression was conducted in cotyledons of living NHCC. Briefly, CDS‐inserted *pCambia1306* was introduced into *Agrobacterium tumefaciens* GV3101. After cultivating overnight, *Agrobacterium tumefaciens* were resuspended using the infiltration medium (0.5% sucrose, pH5.6 and 10 mm 2‐Morpholinoethanesulphonic acid, 10 mm MgCl_2_, 200 µm acetosyringone, and 40 µL L^−1^ Silwet L‐77) with OD_600_ at 0.2–0.3. Cotyledons were infiltrated with a blunt‐end syringe, and the *Agrobacterium tumefaciens*‐infiltrated NHCC plants were cultivated under light conditions for gene overexpression.

### Electrophoretic Mobility Shift Assay (EMSA)

Protein was expressed by virtue of a *pMAL‐c5g* plasmid. Gene‐inserted *pMAL‐c5g* plasmid was introduced into *Escherichia coli* Rosetta DE3, and the *Escherichia coli* Rosetta DE3 cells were cultivated in a shaker with a temperature at 16 °C for overnight protein expression. Expressed proteins were purified with the Anti‐MBP Magnetic Beads (NEB). LightShift EMSA Optimization and Control Kit (ThermoFisher Scientific) was used for detecting the protein‐DNA interaction. A 15 µL protein‐DNA reaction mixture contains 1.5 µL 10 × binding buffer, 2 µg proteins, 1 µL biotin‐labeled oligonucleotides (50 fM), 0.5 µg Poly (dI·dC), competitive oligonucleotides and deionized H_2_O. After a 30‐min incubation at room temperature, the reaction mixture was further electrophoresed with a 5% polyacrylamide gel, and then transferred to a positively charged nylon membrane for UV crosslinking. The Biotin signal was examined using a Chemiluminescent Nucleic Acid Detection Module (ThermoFisher Scientific) with the help of the chemiluminescence image system (Tanon5200).

### Chromatin Immunoprecipitation Assay (ChIP)

Gene overexpression was conducted in cotyledons of living NHCC as described above. After undergoing 2‐day postgene overexpression, NHCC cotyledons were harvested and immersed into 1% formaldehyde vacuuming for 30 min, subsequently, adding glycine to the final concentration at 125 mm and further vacuuming for 10 min to terminate cross‐linking. ChIP was conducted using an EpiQuik Plant ChIP Kit (Epigentek) with anti‐GFP antibody (TransGen Biotech) for immunoprecipitation reaction. The final collected DNA solution was used for qPCR analysis to calculate the protein‐DNA binding capacity. *ACTIN2* was used to normalized the enrichment efficiency, and fragments on CDS were used as the negative control. Relative fold enrichments were calculated as the ratio of those measured in *p35S::EIN3A‐GFP* over those in *p35S:: GFP* (CK). Primers for qPCR are listed in Table S1 (Supporting Information).

### Virus‐Induced Gene Silencing (VIGS) in NHCC

VIGS was conducted in virtue of a turnip yellow mosaic virus‐derived construct *pTY‐S* (*pTY*) in NHCC. *pTY‐CMT2* and *pTY‐EIN3A* constructs were obtained by inserting self‐hybridized palindromic oligonucleotides (5′‐TTGACTGTGATGACATGAAGCGTTCTAGCAGTAATAAACGCGTTTATTACTGCTAGAACGCTTCATGTCATCACAGTCAA‐3′ for *CMT2* silence, 5′‐GTGATCCTCCTCAGAGAAGGTTTCCTTTGGAGAAAGGTGTACACCTTTCTCCAAAGGAAACCTTCTCTGAGGAGGATCAC‐3′ for *EIN3A* silence) into the SnaBI site of *pTY*. Oligonucleotides were synthesis and inserted into *pTY* by the GenScript Biotech company (Nanjing). Virus inoculation was conducted following the previous protocols through particle bombardment using a High‐Pressure Gas Gene Gun.^[^ ^35^ ^]^ Alternatively, virus inoculation was conducted through vacuum infiltration. Briefly, NHCC seeds were germinated on wet filer paper, after cotyledons broke out of the seed shells, seedlings were cut to generate wound and then vacuumed with 500−1000 ng µL^−1^ *pTY‐CMT2* or *pTY‐EIN3A* plasmid solution for 30 min, *pTY* inoculated samples were used as the control. Before analyzing gene silencing‐induced physiological and molecular alterations, cotyledons from the living plant were primarily harvested to examine the silencing efficiency via qPCR.

### Yeast Two‐Hybrid (Y2H) Assay

*CMT2* and *EIN3A* were respectively inserted into *pGAD‐T7* and *pGBK‐T7* plasmids. Constructed *pGAD‐T7* and *pGBK‐T7* plasmids were co‐introduced into Y2H‐gold yeast cells. Plasmid‐introduced Y2H‐gold yeast cells were first cultivated in SD/‐Leu/‐Trp medium, after then resuspended in sterile water. The cell suspension with OD_600_ at 0.5 (or diluted suspension) was further dotted on solid SD/−Leu/−Trp and SD/−Leu/−Trp/−Ade/−His mediums. *pGADT7‐CMT2* + *pGBKT7*, and *pGADT7* + *pGBKT7‐EIN3A* introduced Y2H‐gold yeast cells were used as negative controls.

### Statistical Analysis

All experiments were carried out with at least three independent biological replicates. Data were presented as means ± SD. Data analyses were performed using IBM SPSS Statistics (ver. 24.0). Differences among treatments were analyzed with one‐way ANOVA followed by Duncan's multiple range test to compare treatments (or a Student's *t*‐test when only two treatments were compared). Asterisks indicate significant differences relative to the control (^*^ *p* < 0.05, ^**^ *p* < 0.01, ^***^ *p* < 0.001; Student's *t*‐test). Different letters indicate a significant difference among treatments (*p* < 0.05; one‐way ANOVA followed by Duncan's multiple range test).

## Conflict of Interest

The authors declare no conflict of interest.

## Author Contributions

D.Z. and Y.L. contributed equally to this work. D.Z., B.K., H.Z., and G.R. designed the experiments and wrote the paper. D.Z. and Y.L. performed the experiments and analyzed the data. H.W. contributed to raw sequence data analysis and figure presentation. X.L., L.M., L.G., H.H., and X.W. contributed to data analysis and creation of plant materials. All authors read and approved the final manuscript.

## Supporting information



Supporting Information

Supplemental Table 1

## Data Availability

The data that support the findings of this study are available in the supplementary material of this article.
