# Supplementary material for: An Antagonism Between Ethylene Signaling and DNA Methylation Orchestrates the Progression of Leaf Senescence in Non‐heading Chinese Cabbage
Source: Adv Sci (Weinh). 2025 Jun 23;12(35):e14954. doi: 10.1002/advs.202414954 (PMC12463009; doi:10.1002/advs.202414954)
Supplement: Supplementary file 1 — Supporting Information [file ADVS-12-e14954-s001.docx]

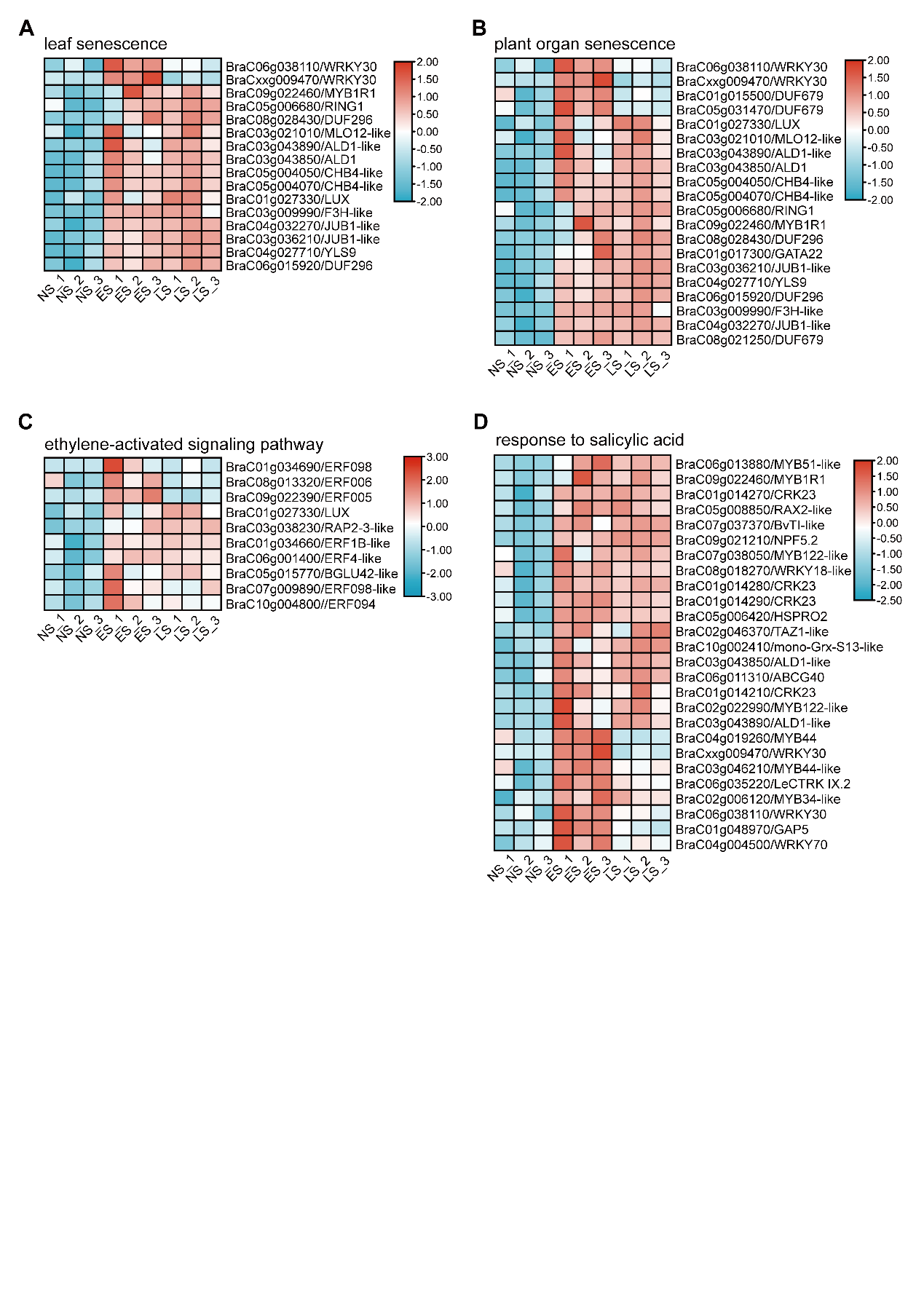


**Figure S1. Expression profile of phytohormone and senescence related genes in cluster 1**

(A) Heatmap of leaf senescence pathway enriched genes. (B) Heatmap of plant organ senescence pathway enriched genes. (C) Heatmap of ethylene-activated signaling pathway enriched genes. (D) Heatmap of response to salicylic acid pathway enriched genes. DEGs were defined with a threshold setting at the absolute value of log_2_FoldChange ≥ 1, and *P*-value < 0.01.


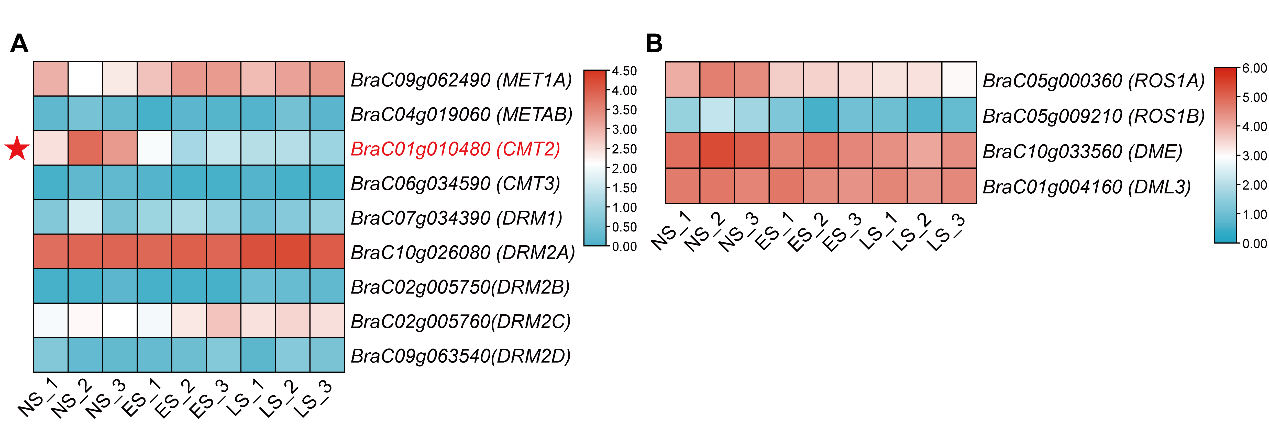


**Figure S2. Expression profile of DNA methylation-related genes during leaf senescence in NHCC**

**(A)** A heatmap showing the expression profiles of DNA methyltransferase encoding genes during leaf senescence in NHCC. **(B)** A heatmap showing the expression profiles of DNA demethylase encoding genes during leaf senescence in NHCC. Data were presented as the log_2_ of FPKM values from transcriptome data.


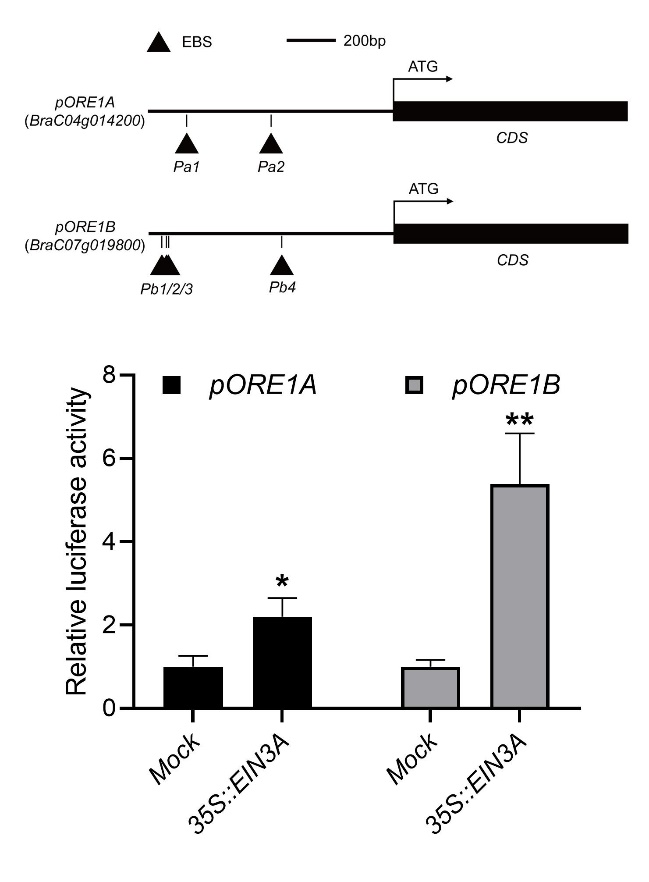


**Figure S3. Dual-luciferase analysis of the effect of EIN3A on the activity of *ORE1s’* promoters.**

*pORE1s*::*FfLUC* were co-introduced into tobacco with *p35S::EIN3A* via the mediation of *Agrobacterium tumefaciens*, with empty vector-transfected samples being used as the controls. Data are means ± SD (n = 3 biological replicates), **P* < 0.05, ***P* < 0.01, (*t*-test).


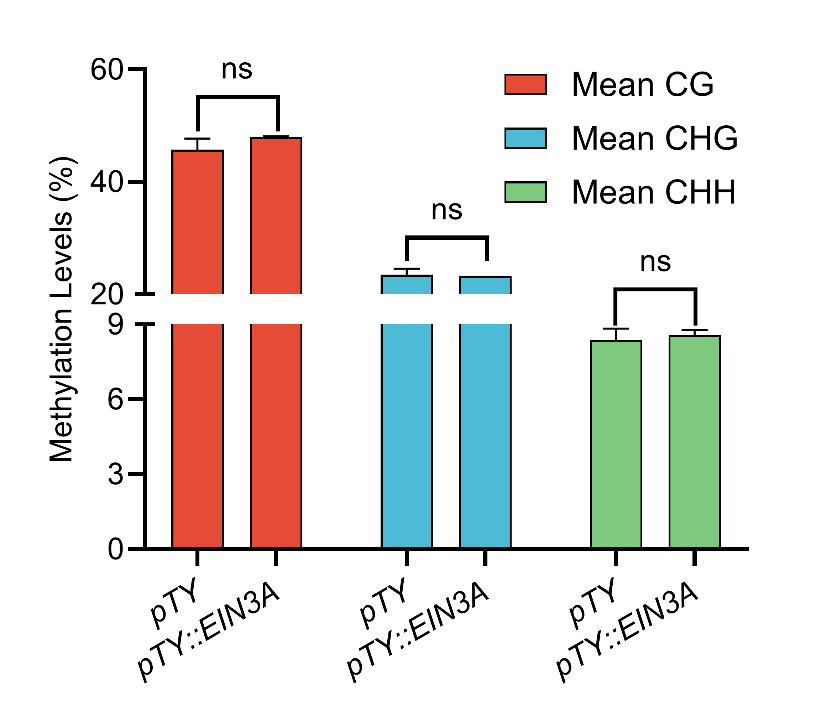


**Figure S4. Genome-wide methylation levels of CG, CHG, and CHH contexts in *pTY* and *pTY::EIN3A* plants**

One-month old NHCC plants were subjected to gene silencing, and WGBS was conducted five days post VIGS.


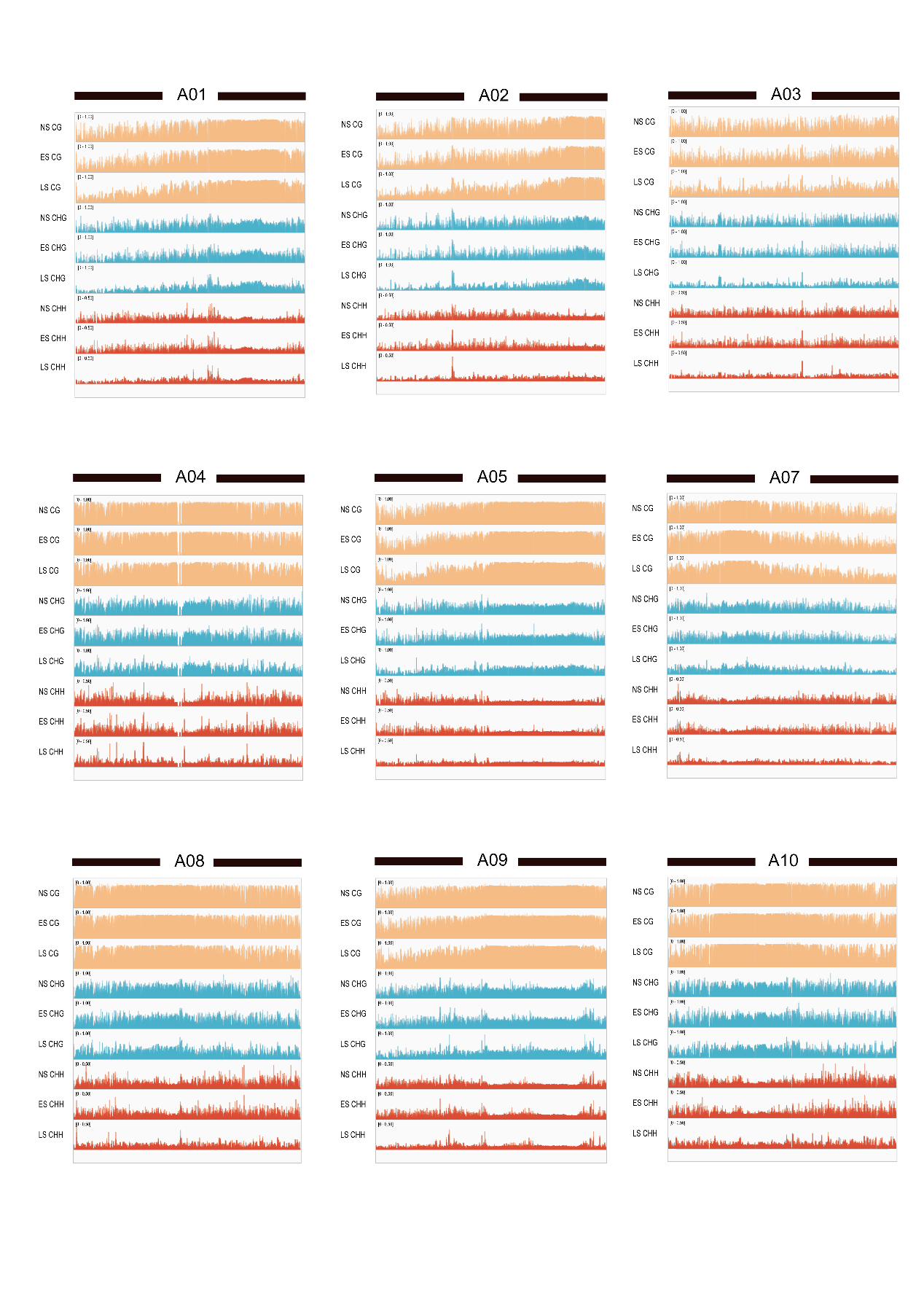


**Figure S5. IGV browser showing the DNA methylation level of A01−A05, A07−A10 chromosomes in NS, ES, LS leaves.**


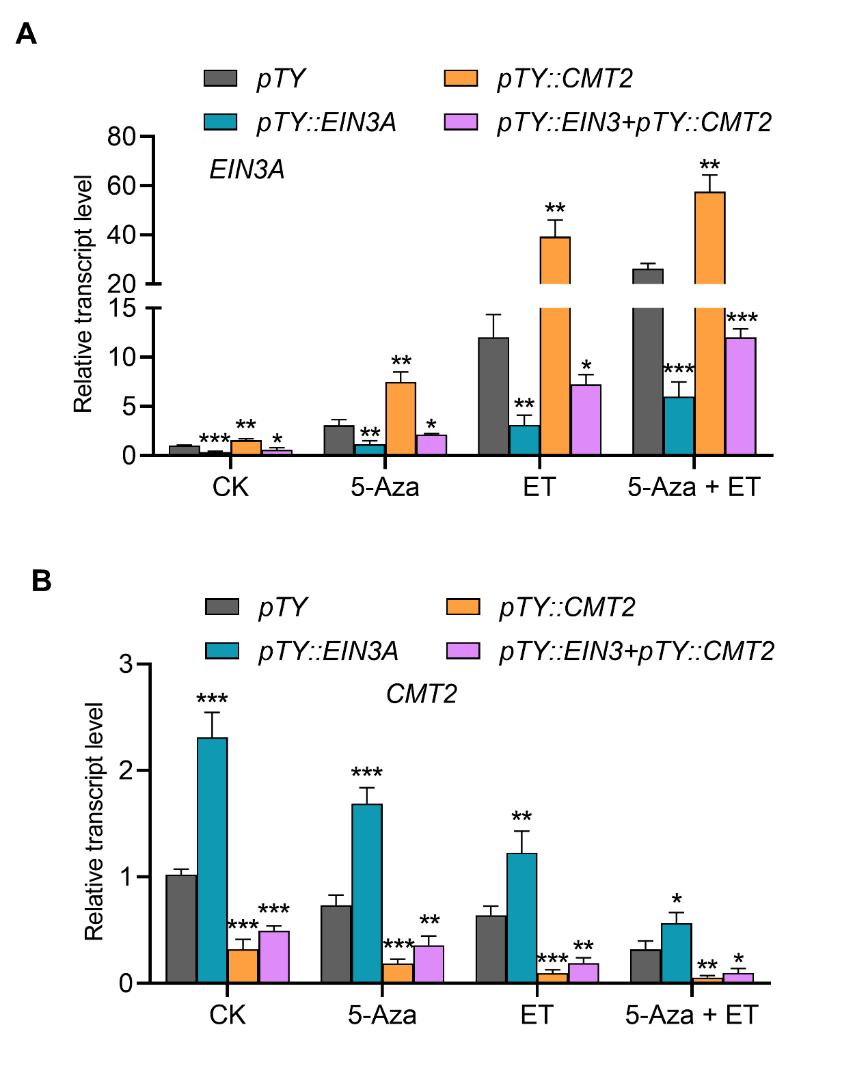


**Figure S6. Relative transcript levels of *EIN3A* and *CMT2* in EIN3A- and CMT2-silenced NHCC leaves under chemical treatments**

The expression of *EIN3A* and *CMT2* in the control (*pTY*) sample was arbitrarily set to 1. Relative transcript levels were calculated as the ratio of those leaves over the control *pTY* leaves. Data are means ± SD (n = 3 biological replicates). **P* < 0.05, ***P* < 0.01, ****P* < 0.001 (*t*-test).


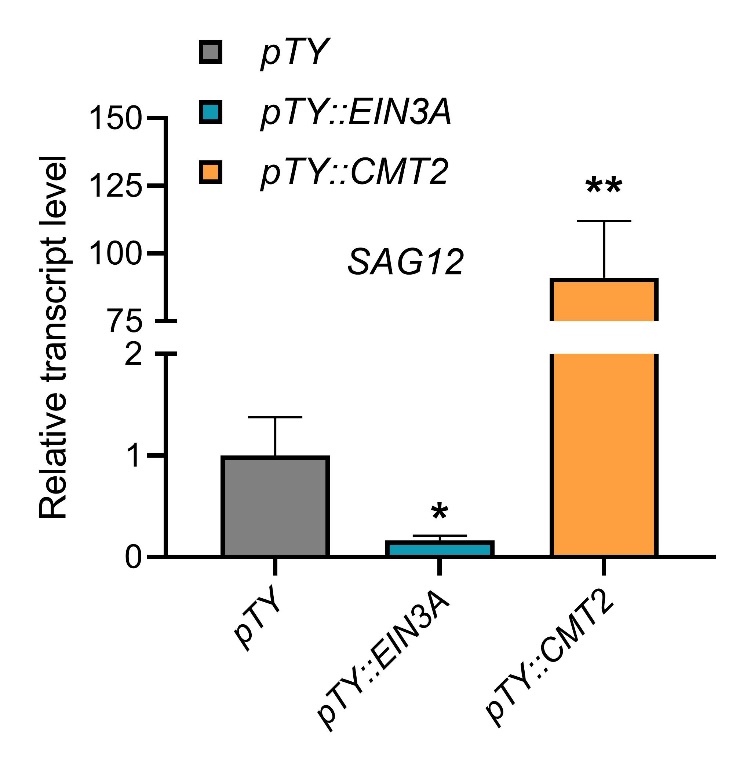


**Figure S7. Relative transcript levels of *SAG12* in the 1^st^ and 2^nd^ true leaves of *CMT2*- and *EIN3A*-silenced NHCC (Caixin) plants after 50-day growth**

Relative transcript levels were calculated as the ratio of those measured in *pTY::EIN3A* or *pTY::CMT2* leaves over those in *pTY* leaves. Data are means ± SD (n = 3 biological replicates), **P* < 0.05, ***P* < 0.01, ****P* < 0.001 (*t*-test).


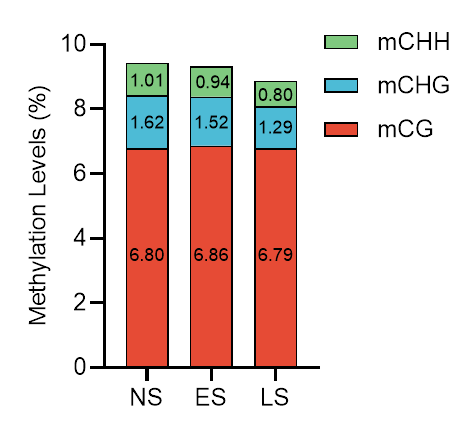


**Figure S8. Methylation level of 1,219 upregulated *SAGs* in CG, CHG, and CHH contexts.**

DEGs were defined with a threshold setting at the absolute value of log_2_FoldChange ≥ 1, and *P*-value < 0.01. The *Arabidopsis thaliana* *SAGs* list was obtained from https://ngdc.cncb.ac.cn/lsd, NHCC *SAGs* were obtained through protein sequence alignment with *Arabidopsis thaliana* *SAGs*.


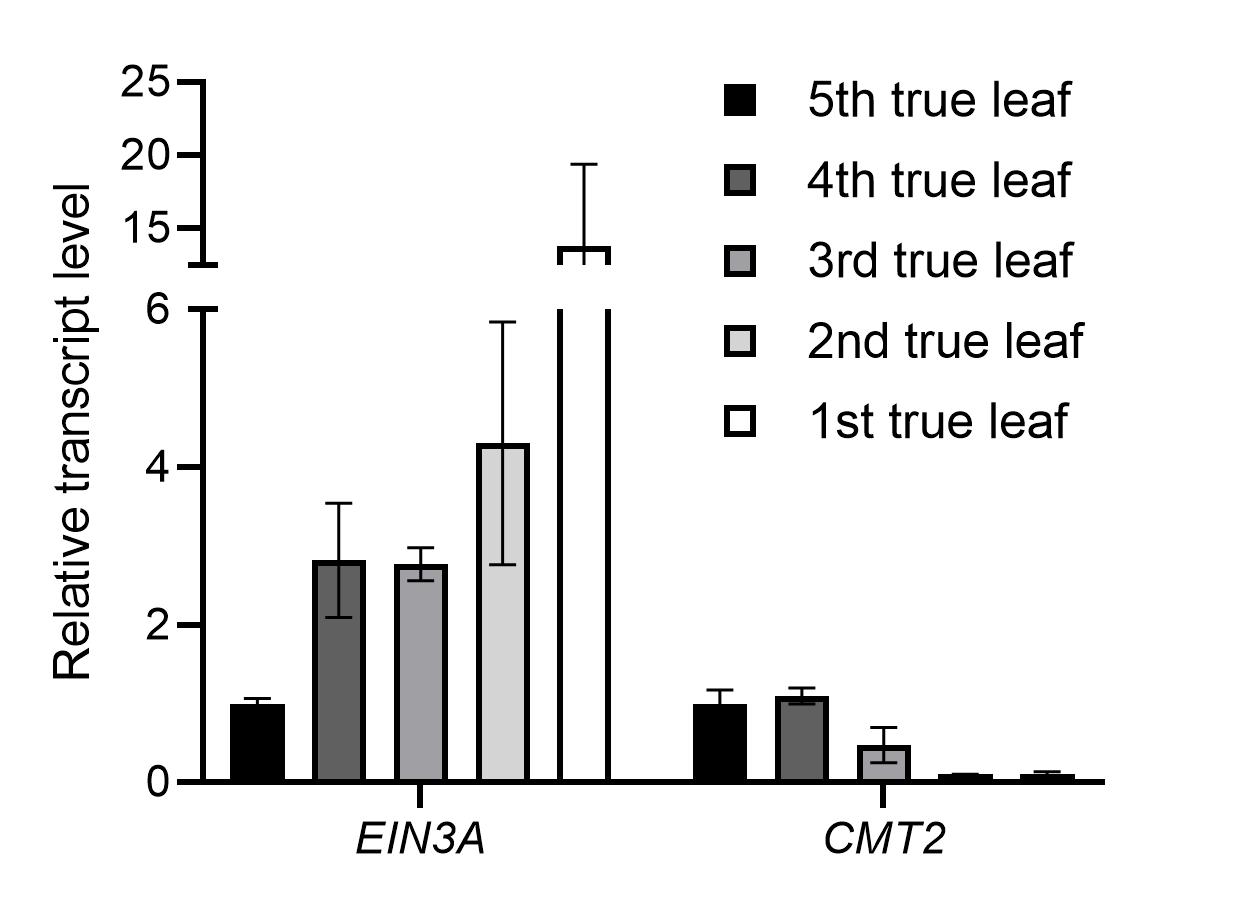


**Figure S9. Expression level of *EIN3A* and *CMT2* in different age true leaves in NHCC**

Two-month-old NHCC plants were used to examination. The true leaves from bottom to up of NHCC plant were assigned as the 1st–5th true leaves, respectively. Data are means ± SD (*n* = 2 biological replicates). Primers sequences are listed in Table S1.


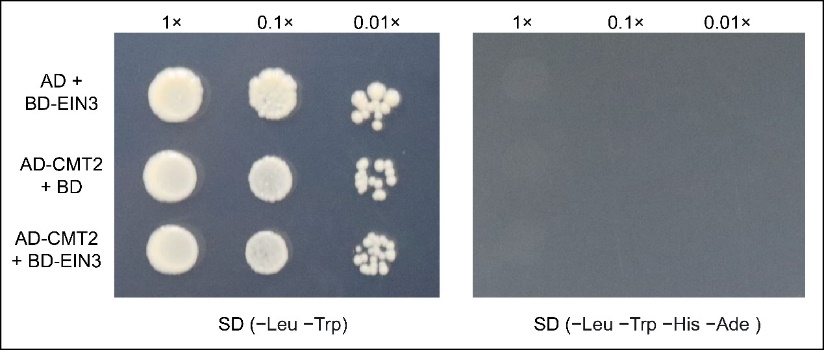


**Figure S10. Physical interaction analysis of EIN3 and CMT2 in Y2H assay.**

AD-CMT2 and BD-EIN3 plasmids were co-transferred into yeast strain Y2H-gold, and the detected clone was grown and screened on a quadruple dropout medium (SD –Leu, –Trp, –Ade, –His). AD + BD-EIN3 and AD-CMT2 + BD transformed yeast cells were used as negative controls.
